# Comparison of Intravenous and Nebulized Dexmedetomidine for Surgical Field Visualization in Functional Endoscopic Sinus Surgeries: A Randomized Controlled Study

**DOI:** 10.7759/cureus.104742

**Published:** 2026-03-05

**Authors:** Hemanthkumar Tamilchelvan, Mayank Gupta, Jyoti Kanwat, Gopal Jalwal, Vaibhav Saini

**Affiliations:** 1 Department of Anesthesiology, All India Institute of Medical Sciences, Bathinda, IND; 2 Department of Otorhinolaryngology, All India Institute of Medical Sciences, Bathinda, IND

**Keywords:** functional endoscopic sinus surgery (fess), laryngoscopy response, nebulized dexmedetomidine, postoperative nausea and vomiting (ponv), postoperative sore throat

## Abstract

Introduction: Functional endoscopic sinus surgery (FESS) requires optimal surgical field visualization. This study was conducted to compare the efficacy of intravenous (IV) and nebulized dexmedetomidine on surgical field visualization in FESS.

Methods: The study population (n = 114) was randomly divided into group A (nebulized dexmedetomidine), group B (IV dexmedetomidine), and group C (normal saline). The primary outcome was to compare the efficacy of IV and nebulized dexmedetomidine on surgical field visualization. The secondary outcomes were to evaluate the hemodynamic response to laryngoscopy and intubation, and to compare blood loss, sedative effects, postoperative sore throat (POST), and postoperative nausea and vomiting (PONV).

Results: Both groups A and B provided optimal surgical field visualization, but group A maintained it for a longer duration (p < 0.05). After laryngoscopy, both groups A and B attenuated the response; however, the increase in heart rate was significantly lower in group B. There was no significant difference in blood pressure between groups A and B (p > 0.05). Both groups A and B reduced blood loss, reduced POST, and reduced PONV (p > 0.05). Group B was better at preventing a fall in postoperative hemoglobin (p = 0.009) and hematocrit (p = 0.006). Group B produced more sedation than group A, although not clinically significant (p > 0.05).

Conclusions: Both IV and nebulized dexmedetomidine at 1 mcg/kg provided optimal surgical field visualization, attenuated the response to laryngoscopy and intubation and reduced blood loss, POST, and PONV. However, nebulized dexmedetomidine provided surgical field visualization for a longer duration, and IV dexmedetomidine better prevented the postoperative hemoglobin and hematocrit reduction.

## Introduction

Functional endoscopic sinus surgery (FESS) is a minimally invasive surgical approach to the sinuses that preserves mucociliary clearance and normal anatomy [1,2]. However, bleeding at the surgical site can significantly impair the operative field visibility, increasing operative risks and prolonging surgery duration [3,4]. Various anesthetic techniques to mitigate capillary bleeding and thereby optimize the field of vision during surgery have been explored. Controlled hypotension is one such technique that enhances visualization and illumination, improving the surgical field [5]. It is defined as a reduction of the systolic blood pressure (SBP) to 80-90 mmHg, a reduction of mean arterial pressure (MAP) to 50-65 mmHg, or a 30% reduction of baseline mean arterial blood pressure [5]. There are numerous methods of inducing deliberate controlled hypotension, which can be broadly classified into physiologic and pharmacologic techniques [5]. Pharmacological agents are the most reliable method [5,6]. An ideal hypotensive anesthetic agent is one that has easy administration, exhibits a shorter time of onset of action, allows meticulous dose control, ensures rapid elimination, and avoids unwanted side effects like hemodynamic instability [6]. Various agents have been tried, but none is ideal and has been found to have some limitations [4-7].

Dexmedetomidine, a potent and highly selective alpha2 agonist, has emerged as a promising hypotensive agent in FESS [8]. This drug possesses sedative, hypnotic, analgesic, anxiolytic, antinociceptive, and sympatholytic properties with no respiratory depression [9-11]. It has been confirmed that administering dexmedetomidine intravenously before surgery can effectively blunt the response to the laryngoscopy [12]. Nebulized dexmedetomidine offers an alternative to the intravenous (IV) route of administration because the drug that gets absorbed following nebulization gets deposited in the nasal mucosa with the bioavailability of 65%, buccal mucosa with the bioavailability of 82%, as well as the respiratory mucosa [13]. Numerous studies have compared the efficacy of IV and nebulization routes of dexmedetomidine on visualizing the surgical field in FESS separately [4,14,15]. Some studies have also compared the IV and nebulization routes of dexmedetomidine to assess the responses elicited during laryngoscopy and intubation [16,17]. However, to the best of our knowledge, there is no study comparing the efficacy of both routes of dexmedetomidine for surgical field visualization in FESS. Our study evaluated the efficacy of IV and nebulized dexmedetomidine as premedication for surgical field nebulization during FESS.

## Materials and methods

This prospective double-blind randomized controlled study was conducted from 2022 to 2024 in the All India Institute of Medical Sciences, Bathinda, after approval from the institutional ethics committee and Clinical Trial Registry of India registration (registration number CTRI/2023/05/052754 dated May 17, 2023). Sample size was computed from the difference between two proportion formulas, considering Boezaart’s bleeding score from the previous study done by Parvizi and Haddadi to be 114 patients (38 in each group) with a confidence interval of 99%, power of 80%, and attrition rate of about 10% [4]. This study included adult patients aged 18 to 60 years, American Society of Anesthesiologists Physical Status (ASA PS) I or II, scheduled for FESS under general anesthesia. Patients not giving consent, pregnant patients (dexmedetomidine crosses the placenta and may cause fetal bradycardia or sedation), those allergic to any of the study drug, and those with moderate-severe cardiovascular (dexmedetomidine causes dose-dependent bradycardia and hypotension due to central sympatholysis), respiratory (compromised pulmonary reserve - chronic obstructive pulmonary disease, restrictive lung disease can worsen perioperative hypoxemia), renal or hepatic impairment (dexmedetomidine undergoes hepatic metabolism and renal excretion. Impairment of these systems prolongs drug clearance), anticipated difficult airway (dexmedetomidine can cause sedation consequently hypoxia while intubation), and those with bleeding diathesis (FESS involves mucosal manipulation and potential bleeding. Coagulopathy or antithrombotic medication use increases perioperative bleeding risk, interfering with surgical field visualization) were excluded [17].

After preanesthetic evaluation and after considering the inclusion and exclusion criteria, adult patients scheduled for FESS were recruited for the study. Informed written consent was taken in the patient’s own vernacular language. Recruited patients were randomly allocated into three equal groups (Group A: nebulized dexmedetomidine; Group B: IV dexmedetomidine; and Group C: control group) by a computer-generated list of random numbers. Sequentially numbered, sealed opaque envelopes were used for allocation concealment. Each envelope contained the assigned group code according to the randomization list. The envelopes were opened only after the patient recruitment was done. To blind the participants, the ENT surgeon, the anesthesiologist administering the drugs and recording the vitals, and the researcher were unaware of the group allocation. Study drugs were prepared by a different anesthesiologist who was not involved in further patient management or data collection. The study solutions (nebulized or IV) were identical in appearance and volume (5 mL), regardless of whether they contained dexmedetomidine or saline.

All patients were kept nil per oral as per guidelines prior to the surgery. Baseline hemoglobin and hematocrit values were noted. On arrival at the preoperative area, an IV line was secured on the nondominant hand, and IV crystalloids at the rate of 100 mL/hour were started. ASA's standard monitors were attached, and baseline parameters, i.e., heart rate (HR), SBP, diastolic blood pressure (DBP), MAP, and oxygen saturation (SpO_2_), were noted.

Dexmedetomidine 100 μg/mL was loaded into an insulin syringe, with each unit constituting 2.5 μg. Dexmedetomidine 1 μg/kg was loaded in a 5 mL syringe diluted to 5 mL with normal saline; 5 mL of normal saline either IV or nebulization was used as a placebo. The drugs were loaded into identical 5 mL syringes by an anesthesiologist not included further in the study. Syringes were labeled as the IV or nebulization study drugs. In group A, dexmedetomidine nebulization was administered at a dose of 1 μg/kg 45 minutes prior to intubation, and IV normal saline was administered 20 minutes prior to intubation. In group B, nebulization with normal saline 45 minutes prior to intubation and IV dexmedetomidine 1 μg/kg, 20 minutes prior to intubation, was administered. In group C, nebulization with normal saline 45 minutes prior to intubation and IV normal saline 20 minutes prior to intubation was administered.

In the preoperative area, nebulization of the study drug was performed 45 minutes prior to intubation, and hemodynamic parameters, such as HR, SBP, DBP, MAP, and SpO_2_, were recorded at five-minute intervals until 20 minutes after the start of nebulization. The patient was then transferred to the operating room, and standard ASA monitors were attached. The IV study drug was administered 20 minutes prior to intubation, and hemodynamic parameters were recorded at five-minute intervals for 15 minutes after IV administration. After three minutes of preoxygenation with 100% oxygen and premedication with opioids, induction was done with titrated doses of propofol (1.5-2.5 mg/kg). After loss of response to verbal contact, vecuronium 0.1 mg/kg was administered as a muscle relaxant. Endotracheal intubation was performed with an appropriately sized tube, and the airway was secured. Patients requiring two or more attempts at intubation were excluded from the study. Hemodynamic parameters were noted at an interval of one minute for the first 5 and 10 minutes after intubation.

A variation of up to 20% from the baseline vitals was accepted. If HR increased by more than 100 beats/minute or blood pressure increased by more than 20% from baseline vitals, a propofol infusion of 50-150 mg/kg/minute was used as a rescue to maintain vitals. HR below 50 beats/minute was considered bradycardia and was treated with IV atropine 0.6 mg. Hypotension was defined as SBP less than 90 mm Hg or a fall of more than 20% from baseline and was treated with IV crystalloid fluid 200 mL over about 10 minutes; if it did not respond, IV mephentermine 6 mg was administered. Surgical field visualization during FESS was assessed using a six-point scale [18,19]: 5: massive uncontrollable bleeding, 4: bleeding heavy but controllable that significantly interfered the surgical field, 3: moderate bleeding that moderately compromised surgical field, 2: moderate bleeding, a nuisance but without interference, 1: bleeding, so mild that it was not even a surgical nuisance, and 0: no bleeding, virtually bloodless field [18,19]. Grading was requested from the operating surgeon at 30-minute intervals until the end of the surgery.

The amount of blood loss during the surgery was assessed using a dedicated, graduated suction canister for each patient to collect intraoperative blood and irrigation fluids: Blood loss (mL) = Total suction volume - Irrigation fluid volume. Standard-size surgical mopping pads were used throughout all procedures.

Each dry mop was weighed before use, and the used mop was weighed immediately after surgery. The difference in weight corresponded to the amount of blood absorbed, assuming 1 g = 1 mL of blood. The total blood absorbed by all mops was then added to the suction blood loss to calculate the total intraoperative blood loss.

At the end of the surgery, IV ondansetron 0.1 mg/kg was given, the neuromuscular block was reversed with an appropriate dose of IV neostigmine and glycopyrrolate, and the patient was extubated after regaining complete consciousness. In the postanesthesia care unit, a blood sample for hemoglobin and hematocrit estimation was collected and sent to the laboratory. Postoperative sore throat (POST) and postoperative nausea and vomiting (PONV) were noted at intervals of 0, 1, 2, 6, 12, and 24 hours. POST severity was measured as 0 (no sore throat at the time since operation), 1 (minimal: patient answered in the affirmative when asked about sore throat), 2 (moderate: patient complained of sore throat on his/her own), and 3 (severe: patient is in obvious distress) [20]. PONV severity was measured as 0 (absent), 1 (mild nausea without vomiting), 2 (severe nausea with vomiting; <3 times/day), and 3 (vomiting; >3 times/day) [21].

During the postoperative period, hemodynamic variables, such as HR, SBP, DBP, MAP, and SpO_2_, were also recorded at 0, 1, and 2 hours, and variations in hemodynamic parameters were treated accordingly. The level of sedation was recorded at arrival to the recovery room, 1, 2, 6, 12, and 24 hours later using a modified observer’s assessment of alertness/sedation score [22]. Modified observer’s assessment of alertness/sedation score is a seven-point scale with 6 (agitated), 5 (responds readily to name spoken in normal tone; alert), 4 (lethargic response to name spoken in normal tone), 3 (responds only after name is called loudly and/or repeatedly), 2 (responds only after mild prodding or shaking), 1 (does not respond to mild prodding or shaking), and 0 (does not respond to deep stimulus) [22]. There were no dropouts from the study.

Statistical analysis

The statistical analysis was carried out using IBM Statistical Package for the Social Sciences software, version 24.0 (IBM Corp., Armonk, NY) for Mac. Descriptive statistical analysis was carried out in this study. Results on continuous measurement were presented as mean and standard deviation (min-max) or median (25-75 quartiles), whereas results on categorical measurement were presented in frequency (%). Chi-square test was performed to compare the categorical variables among the three groups. An independent sample t-test was used to compare continuous variables between the two groups. Chi-square test was used for group comparisons of categorical data. When comparing more than two continuous variables with a normal distribution, the repeated measure analysis of variance (ANOVA) test was used. The Kolmogorov-Smirnov test determined the normality of the distribution. The Mann-Whitney U test was used to compare the differences between two independent groups for non-normal distributions. The Kruskal-Wallis test was used to compare more than two groups for nonnormal distribution. p values <0.05 were considered significant for all the statistical tests.

## Results

**Figure 1:**
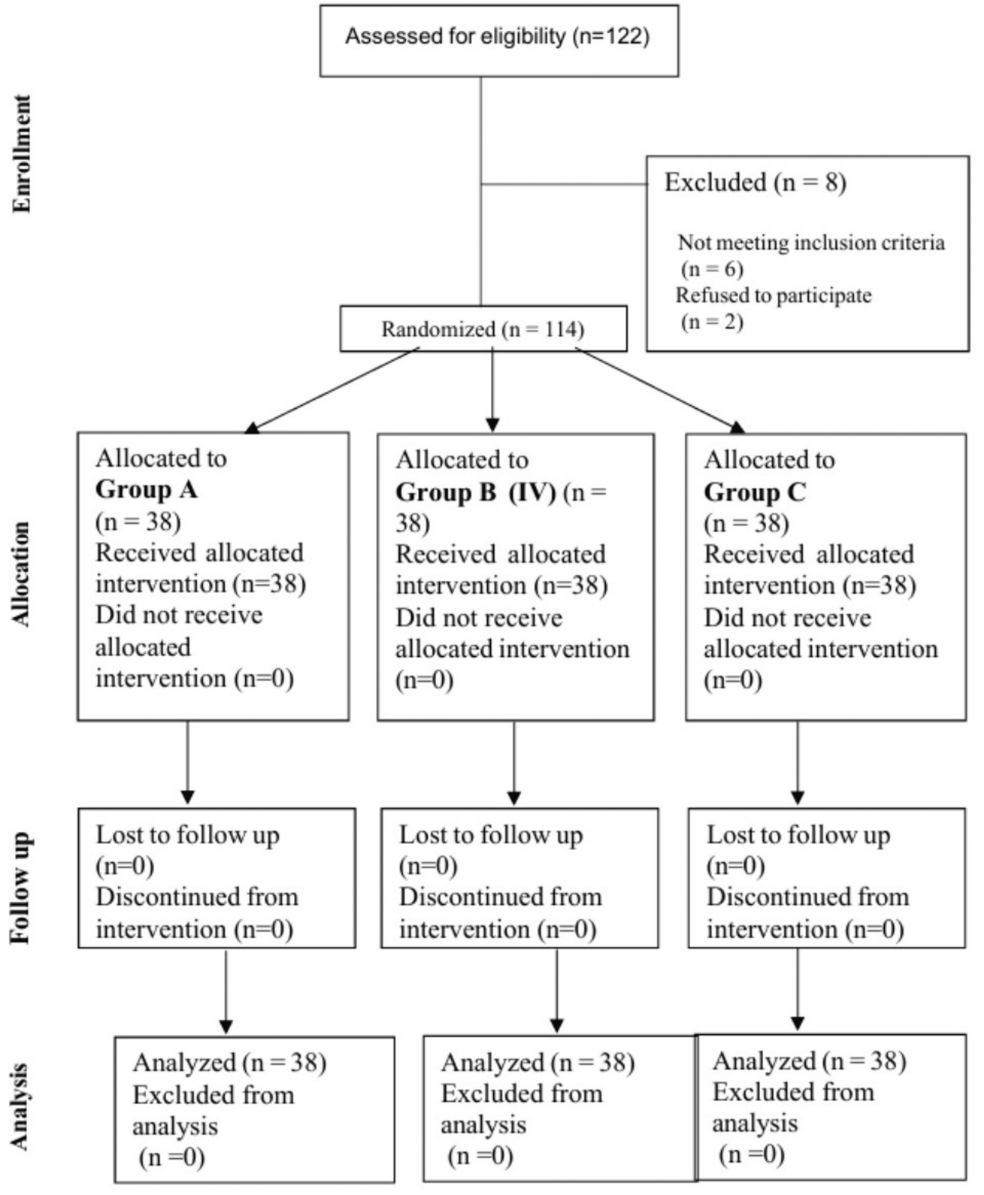
CONSORT flow diagram CONSORT: Consolidated Standards of Reporting Trials

Of 122 patients assessed for eligibility, 114 were recruited and randomly allocated into three equal groups (n = 38) (Figure 1).

Comparison of the surgical field visualization score

Fromme-Boezaart grading was used for the assessment of bleeding and visual assessment of the surgical field, as described in Table 1. Chi-square test was performed. There was a statistically significant difference among the three groups at 30, 60, and 90 minutes. There was no statistically significant difference, and all three groups were comparable at 120, 150, and 180 minutes (Table 1).

**Table 1:** Comparison of surgical field visualization score among three groups ^*^Statistical significance ^†^Chi-square test DOF: degrees of freedom

Surgical field visualization score		Group, n (%)			Total, n (%)	p value				
		A	B	C						
						A vs. B	A vs. C	B vs. C	A vs. B vs. C	
30 minutes	0	15 (39)	22 (58)	3 (8)	40 (35)	0.261^†^ (χ^2^ = 4.01; DOF = 3)	<0.001^†,*^ (χ^2^ = 15.82; DOF = 3)	<0.001^†,*^ (χ^2^ = 20.79; DOF = 3)	<0.001^*,†^ (χ^2^ = 30.80; DOF = 6)	
	1	16 (42)	12 (32)	0 (0)	28 (25)					
	2	5 (13)	4 (11)	22 (58)	31 (27)					
	3	2 (5)	0 (0)	13 (34)	15 (13)					
	4	0 (0)	0 (0)	0 (0)	0 (0)					
	5	0 (0)	0 (0)	0 (0)	0 (0)					
60 minutes	0	17 (45)	23 (61)	2 (5)	42 (37)	0.574^†^ (χ^2 ^= 2.75; DOF = 3)	<0.001^†,*^ (χ^2^ = 7.2; DOF = 3)	<0.001^†,*^ (χ^2^ = 9.34; DOF = 3)	<0.001^*,†^ (χ^2^ = 14.10; DOF = 6)	
	1	15 (39)	11 (29)	8 (21)	34 (30)					
	2	4 (11)	3 (8)	15 (39)	22 (19)					
	3	2 (5)	1 (3)	13 (34)	16 (14)					
	4	0 (0)	0 (0)	0 (0)	0 (0)					
	5	0 (0)	0 (0)	0 (0)	0 (0)					
90 minutes	0	14 (50)	16 (59)	1 (3)	31 (34)	0.598^†^ (χ^2^ = 0.02; DOF = 3)	<0.001^†,*^ (χ^2^ = 17.16; DOF = 3)	<0.001^†,*^ (χ^2^ = 41.16; DOF = 3)	<0.001^*,†^ (χ^2^ = 48.83; DOF = 6)	
	1	12 (43)	9 (33)	8 (23)	29 (32)					
	2	1 (4)	0 (0)	21 (60)	22 (24)					
	3	1 (4)	2 (7)	5 (14)	8 (9)					
	4	0 (0)	0 (0)	0 (0)	0 (0)					
	5	0 (0)	0 (0)	0 (0)	0 (0)					
120 minutes	0	3 (38)	1 (20)	0 (0)	4 (20)	0.788^†^ (χ^2^ = 4.91; DOF = 2)	0.044^*,†^ (χ^2^ = 11.07; DOF = 3)	0.120^†^ (χ^2^ = 43.44; DOF = 3)	0.075^†^ (χ^2^ = 55.87; DOF = 6)	
	1	4 (50)	3 (60)	3 (43)	10 (50)					
	2	0 (0)	0 (0)	4 (57)	4 (20)					
	3	1 (13)	1 (20)	0 (0)	2 (10)					
	4	0 (0)	0 (0)	0 (0)	0 (0)					
	5	0 (0)	0 (0)	0 (0)	0 (0)					
150 minutes	1	1 (50)	0 (0)	1 (50)	2 (40)	0.386^†^ (χ^2^ = 15.31; DOF = 1)	1.000^†^ (χ^2^ = 0.05; DOF = 1)	0.386^†^ (χ^2^ = 0.99; DOF = 1)	0.659^†^ (χ^2^ = 16.06; DOF = 2)	
	2	1 (50)	1 (100)	1 (50)	3 (60)					
	3	0 (0)	0 (0)	0 (0)	0 (0)					
	4	0 (0)	0 (0)	0 (0)	0 (0)					
	5	0 (0)	0 (0)	0 (0)	0 (0)					
180 minutes	1	0 (0)	0 (0)	0 (0)	0 (0)	1.000^†^ (χ^2^ = 0; DOF = 0)	1.000^†^ (χ^2^ = 0; DOF = 0)	1.000^†^ (χ^2^ = 0; DOF = 0)	1.000^†^ (χ^2^ = 0; DOF = 0)	
	2	1 (100)	1 (100)	0 (0)	2 (100)					
	3	0 (0)	0 (0)	0 (0)	0 (0)					
	4	0 (0)	0 (0)	0 (0)	0 (0)					
	5	0 (0)	0 (0)	0 (0)	0 (0)					

To explore further, a pairwise comparison was made using the chi-square test. There was no significant difference between groups A and B at 30, 60, 90, 120, 150, and 180 minutes. On comparing group A and group C, there was a statistically significant difference between the two groups at 30, 60, 90, and 120 minutes. There was no statistically significant difference between group A and group C at 150 and 180 minutes. A comparison was made between groups B and C, and we found a statistically significant difference between the groups at 30, 60, and 90 minutes, whereas there was no statistically significant difference between the groups at 120, 150, and 180 minutes. It has been observed that the nebulized dexmedetomidine produced optimal surgical field visualization compared with the control group for a greater period of time until 120 minutes, while the IV dexmedetomidine group produced only until 90 minutes.

In our study, baseline characteristics such as age, gender, body mass index, and ASA PS grade distribution were comparable among the three groups. Table 1 shows that the nebulized dexmedetomidine produced optimal surgical field visualization compared with the control group for a greater period of time until 120 minutes, while the IV dexmedetomidine group produced only until 90 minutes.

Heart rate

**Figure 2:**
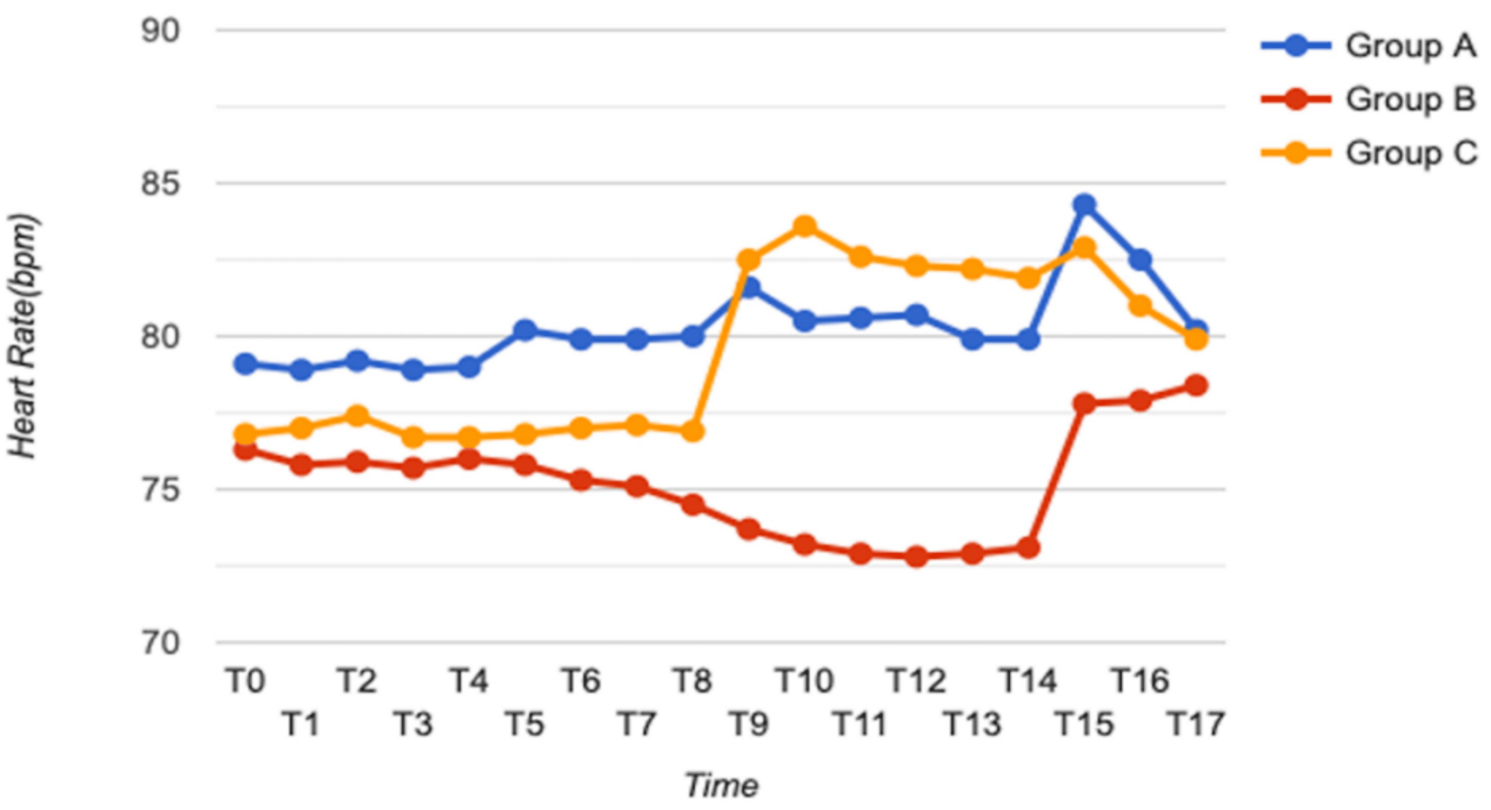
Comparison of heart rate of all three groups

The ANOVA test was performed to compare HRs at different time intervals across three groups (Table 2, Figure 2). There was a statistically significant difference among the three groups at time intervals including 10 minutes after IV, 15 minutes after IV, one minute after intubation, two minutes after intubation, three minutes after intubation, four minutes after intubation, five minutes after intubation, and 10 minutes after intubation. There was no significant difference at baseline and at different time intervals, such as five minutes after nebulization, 10 minutes after nebulization, 15 minutes after nebulization, 20 minutes after nebulization, on shifting to OR, and five minutes after IV. There was no significant difference among the three groups at zero, one, and two hours after extubation.

**Table 2:** Comparison of heart rate of all three groups ^*^p < 0.05 is considered significant ^₤^Independent t-test ^µ^ANOVA test SD: standard deviation; ANOVA: analysis of variance; IV: intravenous

Heart rate in bpm, mean ± SD	Group A	Group B	Group C	p value			
				A vs. B	A vs. C	B vs. C	A vs. B vs. C
Baseline (T0)	79.1 ± 8	76.3 ± 7.2	76.8 ± 6.7	0.122^₤^	0.179^₤^	0.780^₤^	0.351^µ^
5 minutes after nebulization (T1)	78.9 ± 8.3	75.8 ± 7.4	77 ± 6.5	0.088^₤^	0.273^₤^	0.442^₤^	0.390^µ^
10 minutes after nebulization (T2)	79.2 ± 8.7	75.9 ± 7.1	77.4 ± 6.7	0.077^₤^	0.312^₤^	0.362^₤^	0.369^µ^
15 minutes after nebulization (T3)	78.9 ± 7.7	75.7 ± 6.6	76.7 ± 6.7	0.054^₤^	0.191^₤^	0.502^₤^	0.257^µ^
20 minutes after nebulization (T4)	79 ± 7.8	76 ± 6.6	76.7 ± 6.8	0.074^₤^	0.171^₤^	0.658^₤^	0.312^µ^
On shifting to OR (T5)	80.2 ± 9	75.8 ± 6.3	76.8 ± 6.8	0.015^*,₤^	0.063^₤^	0.520^₤^	0.090^µ^
5 minutes after IV (T6)	79.9 ± 8.1	75.3 ± 7	77 ± 6.3	0.009^*,₤^	0.085^₤^	0.255^₤^	0.081^µ^
10 minutes after IV (T7)	79.9 ± 7.8	75.1 ± 7.2	77.1 ± 6.9	0.006^*,₤^	0.097^₤^	0.218^₤^	0.038^*,µ^
15 minutes after IV (T8)	80 ± 8.2	74.5 ± 7.4	76.9 ± 6.1	0.003^*,₤^	0.071^₤^	0.129^₤^	0.022^*,µ^
1 minute after intubation (T9)	81.6 ± 9.8	73.7 ± 7.6	82.5 ± 6.5	<0.001^*,₤^	0.650^₤^	<0.001^*,₤^	<0.001^*,µ^
2 minutes after intubation (T10)	80.5 ± 8.3	73.2 ± 7.6	83.6 ± 6.6	<0.001^*,₤^	0.076^₤^	<0.001^*,₤^	<0.001^*,µ^
3 minutes after intubation (T11)	80.6 ± 8.8	72.9 ± 7.7	82.6 ± 7	<0.001^*,₤^	0.271^₤^	<0.001^*,₤^	<0.001^*,µ^
4 minutes after intubation (T12)	80.7 ± 9.2	72.8 ± 8.2	82.3 ± 7.2	<0.001^*,₤^	0.394^₤^	<0.001^*,₤^	<0.001^*,µ^
5 minutes after intubation (T13)	79.9 ± 8.8	72.9 ± 8.2	82.2 ± 6.9	0.001^*,₤^	0.217^₤^	<0.001^*,₤^	<0.001^*,µ^
10 minutes after intubation (T14)	79.9 ± 8.3	73.1 ± 8.3	81.9 ± 6.7	0.001^*,₤^	0.247^₤^	<0.001^*,₤^	<0.001^*,µ^
0 hour after extubation (T15)	84.3 ± 11.4	77.8 ± 9.1	82.9 ± 7.9	0.008^*,₤^	0.553^₤^	0.011^*,₤^	0.046^µ^
1 hour after extubation (T16)	82.5 ± 6.9	77.9 ± 7.6	81 ± 7.5	0.008^*,₤^	0.368^₤^	0.085^₤^	0.056^µ^
2 hours after extubation (T17)	80.2 ± 7.6	78.4 ± 7.8	79.9 ± 6.6	0.294^₤^	0.860^₤^	0.343^₤^	0.668^µ^

Mean arterial pressure

**Figure 3:**
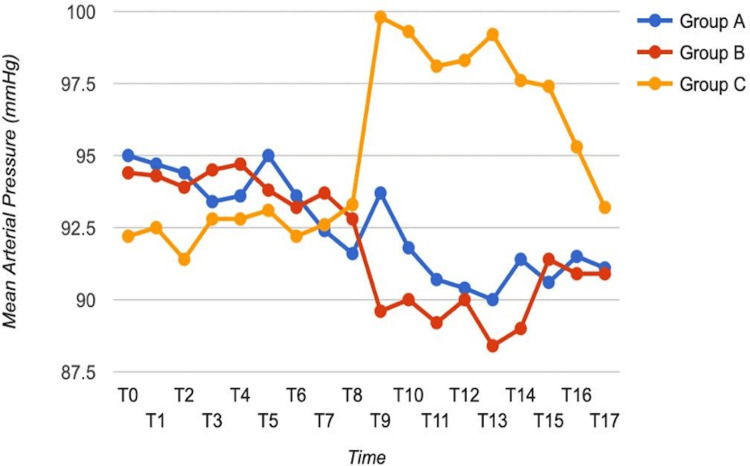
Comparison of mean arterial blood pressure of all three groups

The ANOVA test was performed for the comparison of MAP at different time intervals among three groups (Figure 3, Table 3). There was a statistically significant difference among the three groups at time intervals of 1, 2, 3, 4, 5, and 10 minutes after intubation. There was no significant difference at baseline and at different time intervals, such as five minutes after nebulization, 10 minutes after nebulization, 15 minutes after nebulization, 20 minutes after nebulization, on shifting to OR, five minutes after IV, 10 minutes after IV, and 15 minutes after IV. There was a statistically significant difference among the three groups at zero and one hours after extubation, while there was no significant difference among the three groups at two hours after extubation. Figure 3 shows that both IV and nebulized dexmedetomidine were comparable to each other and attenuated the mean arterial blood pressure in response to laryngoscopy in comparison to the control group.

**Table 3:** Comparison of mean arterial blood pressure of all three groups ^*^p < 0.05 is considered significant ^¥^Independent test ^£^ANOVA test MAP: mean arterial pressure; SD: standard deviation; ANOVA: analysis of variance; IV: intravenous

MAP in mmHg, mean ± SD	A	B	C	p value			
				A vs. B	A vs. C	B vs. C	A vs. B vs. C
Baseline (T0)	95 ± 11	94.4 ± 9.9	92.2 ± 8.2	0.802^¥^	0.215^¥^	0.297^¥^	0.554^£^
5 minutes after neb (T1)	94.7 ± 10.4	94.3 ± 9	92.5 ± 7.7	0.860^¥^	0.289^¥^	0.342^¥^	0.783^£^
10 minutes after neb (T2)	94.4 ± 9.5	93.9 ± 9.1	91.4 ± 7.3	0.835^¥^	0.127^¥^	0.190^¥^	0.276^£^
15 minutes after neb (T3)	93.4 ± 10.1	94.5 ± 9	92.8 ± 8	0.625^¥^	0.773^¥^	0.390^¥^	0.734^£^
20 minutes after neb (T4)	93.6 ± 9.7	94.7 ± 9.9	92.8 ± 8.2	0.625_¥_	0.694^¥^	0.359^¥^	0.628^£^
On shifting to OR (T5)	95 ± 10.3	93.8 ± 9.7	93.1 ± 6.3	0.600^¥^	0.344^¥^	0.727^¥^	0.944^£^
5 minutes after IV (T6)	93.6 ± 10.7	93.2 ± 8.5	92.2 ± 7.4	0.868^¥^	0.511^¥^	0.575^¥^	0.876^£^
10 minutes after IV (T7)	92.4 ± 10.1	93.7 ± 8.8	92.6 ± 6.6	0.555^¥^	0.914^¥^	0.547^¥^	0.802^£^
15 minutes after IV (T8)	91.6 ± 9.1	92.8 ± 8.5	93.3 ± 6.5	0.550^¥^	0.336^¥^	0.751^¥^	0.578^£^
1 minute after intubation (T9)	93.7 ± 10.2	89.6 ± 11.5	99.8 ± 7.4	0.104^¥^	0.004^*,¥^	<0.001^*,¥^	<0.001^*,£^
2 minutes after intubation (T10)	91.8 ± 10.2	90 ± 10.8	99.3 ± 8.8	0.454^¥^	0.001^*,¥^	<0.001^*,¥^	<0.001^*,£^
3 minutes after intubation (T11)	90.7 ± 9.7	89.2 ± 10.1	98.1 ± 6.8	0.518^¥^	<0.001^*,¥^	<0.001^*,¥^	<0.001^*,£^
4 minutes after intubation (T12)	90.4 ± 10.7	90 ± 9.5	98.3 ± 6.4	0.883^¥^	<0.001^*,¥^	<0.001^*,¥^	<0.001^*,£^
5 minutes after intubation (T13)	90 ± 9.5	88.4 ± 9.8	99.2 ± 6.7	0.463^¥^	<0.001^*,¥^	<0.001^*,¥^	<0.001^*,£^
10 minutes after intubation (T14)	91.4 ± 8	89 ± 10	97.6 ± 8.2	0.248^¥^	0.001^*,¥^	<0.001^*,¥^	<0.001^*,£^
0 hour after extubation (T15)	90.6 ± 8.6	91.4 ± 11.3	97.4 ± 8.5	0.725^¥^	0.001^*,¥^	0.010^*,¥^	0.002^*,£^
1 hour after extubation (T16)	91.5 ± 7.6	90.9 ± 10.1	95.3 ± 8.3	0.788^¥^	0.041^*,¥^	0.044^*,¥^	0.034^*,£^
2 hours after extubation (T17)	91.1 ± 7.8	90.9 ± 8.6	93.2 ± 7.9	0.911^¥^	0.263^¥^	0.241^¥^	0.532^£^

Total blood loss and reduction in hemoglobin and hematocrit

Table 4 shows that both IV and nebulized dexmedetomidine reduce total blood loss compared with the control group, and they are comparable to each other, whereas IV dexmedetomidine shows a smaller fall in hemoglobin and hematocrit than the other two groups.

**Table 4:** IV and nebulized dexmedetomidine reducing total blood loss compared with control group, and they were comparable to each other, whereas in IV dexmedetomidine, there is a less fall in hemoglobin and hematocrit compared with other two groups ^*^Statistical significance ^€^ANOVA test SD: standard deviation; ANOVA: analysis of variance; IV: intravenous

Variables, mean ± SD	A	B	C	p value			
				A vs. B	A vs. C	B vs. C	A vs. B vs. C
Total amount of blood loss during surgery in mL	94.7 ± 99.4	75.8 ± 96.4	156.3 ± 96.3	0.402^€^	0.008^*,€^	<0.001^*,€^	<0.001^*,€^
Pre-op hemoglobin	12.8 ± 1.5	13.3 ± 1.1	13 ± 1.2	0.120^€^	0.559^€^	0.265^€^	0.274^€^
Post-op hemoglobin	12.4 ± 1.4	13.1 ± 1.3	12.3 ± 1.2	0.017^*,€^	0.978^€^	0.009^*,€^	0.015^*,€^
Pre-op hematocrit	38.6 ± 3.7	39.2 ± 3	37.9 ± 1.5	0.441^€^	0.280^€^	0.019^*^	0.322^€^
Post-op hematocrit	37.1 ± 2.6	38.1 ± 3	36.6 ± 1.4	0.124^€^	0.290^€^	0.006^*,€^	0.009^*,€^

Postoperative sore throat

**Figure 4:**
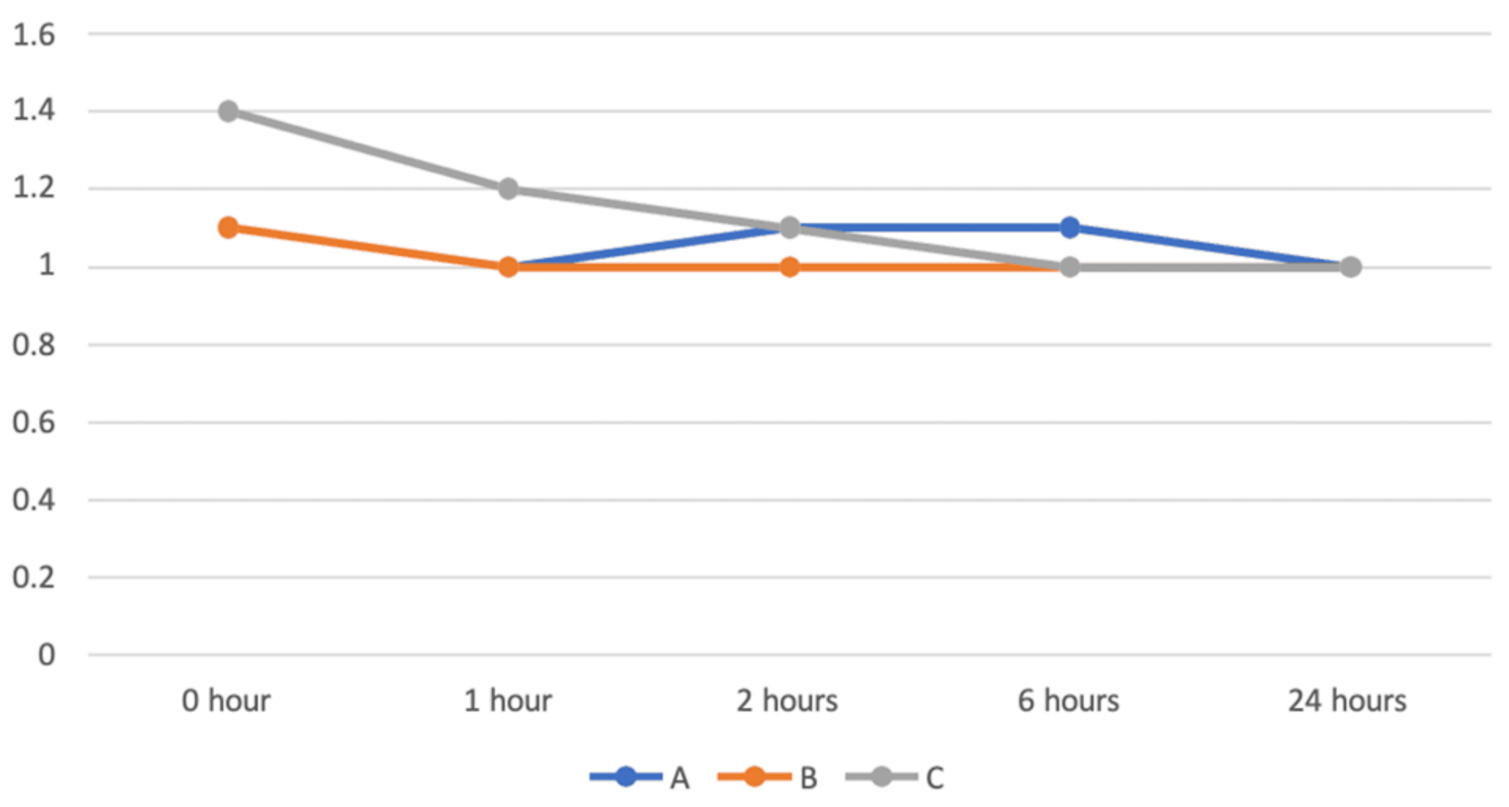
Comparison of postoperative sore throat in all three groups

The POST score was measured at 0, 1, 2, 6, and 24 hours after extubation. The Kruskal-Wallis test was performed for the comparison among groups (Table 5, Figure 4). There was a statistically significant difference among the three groups at 0, 1, and 6 hours after extubation, whereas there was no statistically significant difference among the three groups at two hours and 24 hours after extubation in assessing the POST score. The Mann-Whitney U test was performed for pairwise comparisons. There was no statistically significant difference between groups A and B in assessing the POST score. Comparing groups A and C, there was a statistically significant difference between the two groups at 0 and 1 hours after extubation, while there was no significant difference between the groups at 2, 6, and 24 hours after extubation in assessing the POST score. Comparing groups B and C, there was a statistically significant difference between the two groups at 0 and 1 hours after extubation, whereas there was no significant difference between the groups at 2, 6, and 24 hours after extubation in assessing the POST score.

**Table 5:** Comparison of POST of all three groups ^*^p < 0.05 is considered significant ^¥^Mann-Whitney U test ^µ^Kruskal-Wallis test POST: postoperative sore throat

POST score	A, median (quartiles)	B, median (quartiles)	C, median (quartiles)	p value			
				A vs. B	A vs. C	B vs. C	A vs. B vs. C
0 hour	1 (1,1)	1 (1,1)	1 (1,2)	0.629^¥^ (U = 742.0)	0.003^*,¥^ (U = 517.0)	0.001^*,¥^ (U = 492.0)	<0.001^*,µ^ (H = 16.46)
1 hour	1 (1,1)	1 (1,1)	1 (1,1)	1.000^¥^ (U = 722.0)	0.026^*,¥^ (U = 608.0)	0.026^*,¥^ (U = 608.0)	0.014^*,µ^ (H = 8.61)
2 hours	1 (1,1)	1 (1,1)	1 (1,1)	0.558^¥^ (U = 741.0)	0.398^¥^ (U = 684.0)	0.168^¥^ (U = 665.0)	0.348^µ^ (H = 2.11)
6 hours	1 (1,1)	1 (1,1)	1 (1,1)	0.079^¥^ (U = 779.0)	0.079^¥^ (U = 779.0)	1.000^¥^ (U = 722.0)	0.047^*,µ^ (H = 6.11)
24 hours	1 (1,1)	1 (1,1)	1 (1,1)	0.317^¥^ (U = 741.0)	0.317^¥^ (U = 741.0)	1.000^¥^ (U = 722.0)	0.368^µ^ (H = 2.00)

Both IV and nebulized dexmedetomidine caused a reduction in the incidence of POST in the early postoperative period for one hour and were comparable to each other.

Postoperative nausea and vomiting

**Figure 5:**
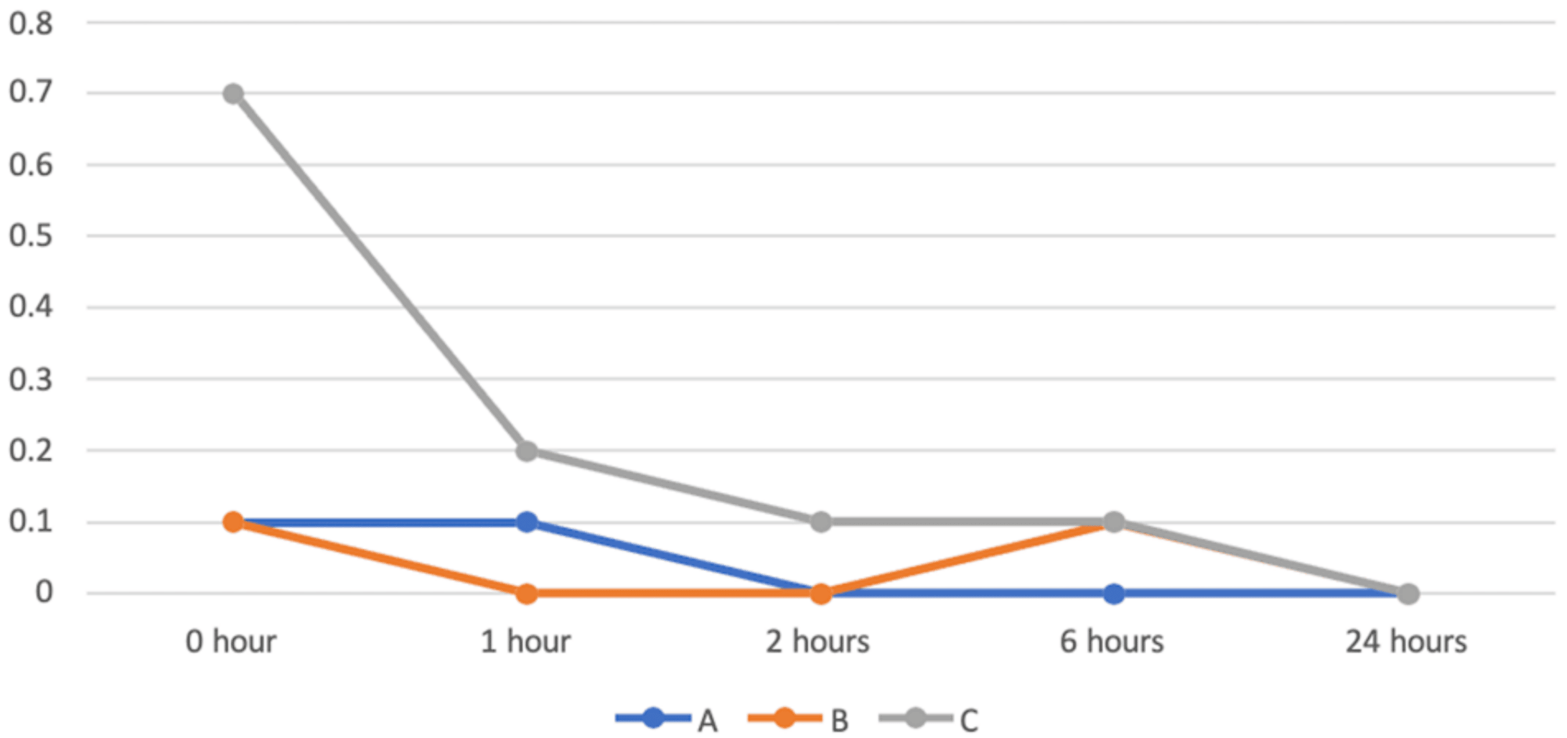
Comparison of postoperative nausea and vomiting in all three groups

The PONV score was measured at 0, 1, 2, 6, and 24 hours after extubation. The Kruskal-Wallis test was performed for the comparison among groups (Table 6, Figure 5). There was a statistically significant difference among the three groups at 0 hours after extubation and one hour after extubation, while there was no statistically significant difference among the three groups at 2, 6, and 24 hours after extubation in assessing the PONV score. The Mann-Whitney U test was performed for pairwise comparisons. Comparing groups A and B, there was no statistically significant difference between groups A and B in assessing the PONV score. Comparing groups A and C, there was a statistically significant difference between the groups at the zeroth hour after extubation, whereas there was no significant difference between the groups at 1, 2, 6, and 24 hours after extubation in assessing the PONV score. Comparing groups B and C, there was a statistically significant difference between the groups at zero and one hours after extubation, whereas there was no significant difference between the groups at 2, 6, and 24 hours after extubation in assessing the PONV score. Both IV and nebulized dexmedetomidine reduced the incidence of PONV immediately after extubation, whereas nebulized dexmedetomidine prevented PONV for up to one hour postoperatively compared with the control group.

**Table 6:** Comparison of PONV of all three groups ^*^p < 0.05 is considered significant ^¥^Mann-Whitney U test ^µ^Kruskal-Wallis test PONV: postoperative nausea and vomiting

PONV score	A, median (quartiles)	B, median (quartiles)	C, median (quartiles)	p value			
				A vs. B	A vs. C	B vs. C	A vs. B vs. C
0 hour	0 (0,0)	0 (0,0)	0 (0,1)	0.979^¥^ (U = 721.0)	<0.001^*,¥^ (U = 462.0)	<0.001^*,¥^ (U = 465.5)	<0.001^*,µ^ (H = 21.97)
1 hour	0 (0,0)	0 (0,0)	0 (0,0)	0.548^¥^ (U = 741.5)	0.090^¥^ (U = 630.5)	0.026^*,¥^ (U = 608.0)	0.038^*,µ^ (H = 6.54)
2 hours	0 (0,0)	0 (0,0)	0 (0,0)	1.000^¥^ (U = 722.0)	0.295^¥^ (U = 683.0)	0.295^¥^ (U = 683.0)	0.418^µ^ (H = 1.75)
6 hours	0 (0,0)	0 (0,0)	0 (0,0)	0.155^¥^ (U = 684.0)	0.317^¥^ (U = 703.0)	0.559^¥^ (U = 741.0)	0.361^µ^ (H = 2.04)
24 hours	0 (0,0)	0 (0,0)	0 (0,0)	1.000^¥^ (U = 722.0)	1.000^¥^ (U = 722.0)	1.000^¥^ (U = 722.0)	1.000^µ^ (H = 0)

Level of sedation

**Figure 6:**
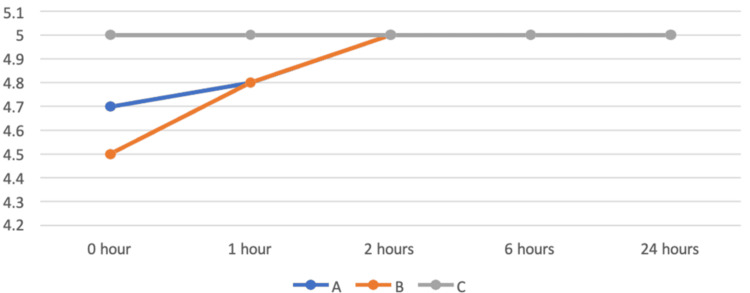
Comparison of sedation in all three groups

Sedation score was measured at 0, 1, 2, 6, and 24 hours after extubation. The ANOVA test was performed for the comparison among groups (Table 7, Figure 6). There was a statistically significant difference among the three groups at one hour after extubation, whereas there was no statistically significant difference among the three groups at 0, 2, 6, and 24 hours after extubation in assessing the level of sedation. An independent t-test was performed for pairwise comparison. Comparing groups A and B, there was no statistically significant difference between groups A and B in assessing the level of sedation. Comparing groups A and C, there was a statistically significant difference between the two groups at zero and one hour after extubation, while there was no significant difference between the two groups at 2, 6, and 24 hours after extubation in assessing the level of sedation. Comparing groups B and C, there was a statistically significant difference between the two groups at zero and one hour after extubation, whereas there was no significant difference between the two groups at 2, 6, and 24 hours after extubation in assessing the level of sedation. Both IV and nebulized dexmedetomidine caused a higher level of sedation compared with the control group, but were comparable to each other's groups.

**Table 7:** Comparison of level of sedation of all three groups ^*^p < 0.05 is considered significant ^¥^Independent t-test ^µ^ANOVA test ANOVA: analysis of variance

Level of sedation	A, median (quartiles)	B, median (quartiles)	C, median (quartiles)	p value			
				A vs. B	A vs. C	B vs. C	A vs. B vs. C
0 hour	5 (4,5)	4.5 (4,5)	5 (5,5)	0.082^¥^	0.004^*,¥^	<0.001^*,¥^	<0.001^*,µ^
1 hour	5 (5,5)	5 (5,5)	5 (5,5)	0.557^¥^	0.011^*,¥^	0.003^*,¥^	0.015^*,µ^
2 hours	5 (5,5)	5 (5,5)	5 (5,5)	1.000^¥^	0.317^¥^	0.317^¥^	0.604^µ^
6 hours	5 (5,5)	5 (5,5)	5 (5,5)	1.000^¥^	1.000^¥^	1.000^¥^	1.000^µ^
24 hours	5 (5,5)	5 (5,5)	5 (5,5)	1.000^¥^	1.000^¥^	1.000^¥^	1.000^µ^

## Discussion

The primary objective of this study was to evaluate and compare the efficacy of IV and nebulized dexmedetomidine in maintaining hemodynamic stability and optimizing the surgical field during FESS. Our results demonstrate that dexmedetomidine, regardless of the route of administration, significantly improves operative field visibility and attenuates the sympathoadrenal response to laryngoscopy and tracheal intubation.

Our results were consistent with those of Sujay et al., who compared the efficacy of IV dexmedetomidine with labetalol in FESS and found that IV dexmedetomidine provided better operative field visibility (p < 0.05) [23]. Studies compared IV dexmedetomidine and esmolol in FESS and found that dexmedetomidine provided ideal surgical field visibility [14,24]. While studying the inflammatory response of intranasal dexmedetomidine in FESS, Tang et al. also inferred that the intranasal dexmedetomidine provided a better surgical field compared with the normal saline group, which was in agreement with our results (p < 0.001) [15].

Niyogi et al. found attenuation of HR and MAP response to laryngoscopy with greater HR reduction in the IV route compared with intranasal dexmedetomidine (p > 0.05) [16]. In a study conducted by Misra et al., there was a lower trend of increase in HR in the nebulized dexmedetomidine group compared with normal saline (p = 0.012), supporting the results of our study [25]. Singh et al. compared IV and nebulized dexmedetomidine’s effect on HR and MAP during laryngoscopy and intubation, finding a fall in HR and MAP in both groups [26]. Our study differed from Kumar et al.’s findings, as they found no significant difference in HR between nebulized dexmedetomidine and normal saline groups. This disparity is possibly due to differing nebulization timings, 10 minutes prior in their study vs. 45 minutes in ours (p < 0.05) [27].

Tang et al. observed that mean blood loss was lower in the intranasal dexmedetomidine group than in the placebo group (p = 0.030) [15]. Sujay et al. compared the efficacy of dexmedetomidine vs. labetalol and found that blood loss was lower in the dexmedetomidine group than in the labetalol group (p < 0.05) [23]. Misra et al. also observed that POST and PONV were reduced with nebulized dexmedetomidine (p > 0.05) [25]. Bajwa et al. observed that the severity of PONV was lower in the dexmedetomidine group, although statistically not significant (p = 0.052) [28]. Bajwa et al. found that Ramsay Sedation scores were significantly higher in the dexmedetomidine group than in the esmolol and nitroglycerine group, consistent with our study results (p < 0.001) [28]. Bafna et al. found that the mean sedation scores were statistically significant and higher in the dexmedetomidine group compared to the clonidine group (p = 0.001) [29]. Shams et al. found that the sedative scores were higher in dexmedetomidine compared with the esmolol group (p < 0.001) [14]. Our results were not in concordance with the study done by Niyogi et al., in which there was a significantly higher sedation score in the IV dexmedetomidine group compared with the intranasal dexmedetomidine group since preoperative sedation levels were measured (p = 0.014) [16].

Strengths of this study

We compared IV and nebulization routes in surgical field visualization, which have not been compared together in a single study. The sample size was substantially adequate to compare different routes of dexmedetomidine. Randomization, blinding, and a placebo group further strengthened our study.

Limitations of this study

However, the limitation of our study is that only adult ASA PS 1 and 2 patients planned for elective surgery in a single institution were included in our study. It is uncertain if our results could be extended to pediatric, obstetric, and geriatric age groups. Cases with anticipated difficult airway were excluded, and the time required for laryngoscopy was not measured. Further multicentric studies with larger sample sizes in different age groups and emergency settings would be required to further strengthen the results of our study.

## Conclusions

Both IV and nebulized dexmedetomidine at 1 mcg/kg provided optimal surgical field visualization, attenuated the response to laryngoscopy and intubation, reduced blood loss, reduced POST, and reduced PONV. However, nebulized dexmedetomidine provided surgical field visualization for a longer duration, and IV dexmedetomidine better prevented the reduction of fall in postoperative hemoglobin and hematocrit. Both nebulized and IV dexmedetomidine were associated with postoperative sedation.
